# Supplementary material for: Communication through autoattractants can enhance and limit collective migration of immune cells
Source: PLoS Comput Biol. 2026 Sep 22;22(9):e1014809. doi: 10.1371/journal.pcbi.1014809 (PMC13626474; doi:10.1371/journal.pcbi.1014809)
Supplement: Supplementary file 1 [file pcbi.1014809.s010.pdf]

Reviewer 1
Reviewer #1: This is a novel and useful computational study of collective cell chemotaxis in the presence of an 'autoattractant' that cells emit and respond to, in addition to common external chemoattractant. The authors simulate intuitive hybrid model in which the cells are discrete traveling agents, while the chemicals are simulated according to reaction-diffusion models. The main finding of the paper is that there are certain optimal regimes in which the autoattractant dynamics have to be fine-tuned in order to maximize the response to the external chemoattractant.

The paper is clearly written, accurate and well fit for PLoS Comp Biol.

We thank the reviewer for their comments

I suggest several minor changes:

1) What I was missing in the text is mechanistic explanations of the results of the numerical experiments. For example, "too much autoattractan --> worse performance" is just stated plainly.
There has to be a simple explanations, like "in this regime cells aggregate too much and then..."
maybe their movement is hindered, or they degrade the external chemoattractant too much, or...
I strongly suggest to write such interpretations after every result of the numerical experiment, and then summarize them in the Discussion.

We have included additional mechanistic explanations of each of the figures, and included more mechanistic explanations in our summary at the start of the discussion.

2) Numbers in Tables 1 and 2 are told to be coming from published data. Need references for these parameters' values, with explanations - was this or that parameter measured, or coming from some indirect inference, or... And I imagine that at least some of these parameters are
from some kinds of fits - basically, need details.

We have added an additional column to each table detailing which values were plausible fits or model assumption, and listing sources for the other parameters. We also go more in depth on diffusion at the start of section 2.6.

3) "Tell your friends:" part of the title is cute, but perhaps not a good idea for a research paper (it would be fine and even welcome for a review)

We have changed the title to “Communication through autoattractants can enhance and limit collective migration of immune cells”

Reviewer 2

This paper builds on the group's work on chemotaxis through self-generated gradients. A new ingredient is introduced: cells not only consume an attractant but also produced an autoattractant.
The paper then determines for which conditions more cells can reach the half-point of the chamber.

I believe the article is not always as clear as it could be. I have some specific comments below. My most serious comment,however, is that it is unclear if this scenario is actually at play in vivo.

The authors acknowledge that autoattractants "would be short-lived, it is inherently difficult to find additional autoattractants in vitro or in vivo: samples collected would be depleted of autoattractant before measurements could be made. New techniques may find autoattractants in many more cell types." This, combined with the assumptions make in the study (constant speed, cells response identical to both attractant and autoattractant, etc.) dampens my enthusiasm for the paper. However, the results are scientifically sound and I urge the authors to make a better argument why this scenario is plausible.

We thank the reviewer for their comments. We have added additional detail in the introduction to show why we think this phenomenon is widespread. In addition, we have clarified in the introduction how a constant speed over 6 second intervals does not necessitate a constant speed over the longer durations over which chemotaxis is usually measured, and we describe in the discussion how the response to autoattractant is freely scaleable, so that an identical response to attractant and autoattractant is not an assumption of the model.

The diffusion constant is taken to be 100 micron^2/s and the timestep is 6s. This means that in one timestep, the attractant can diffuse over a length scale of sqrt(600)~25 micron. I could not find the size of the computational cell but this seems to me very large compared to a, e.g., neutrophil.

We have since corrected an error in the code - implemented diffusion values were actually half, so 50 micron^2/s. We take the area in which the cell can sense attractant to be 30um from the cell center in every direction, to account for variable cell shape and diffusion within our timesteps. This is still fast compared to a neutrophil, but realistic for many attractants, as we now substantiate in table 1 and in the text in section 2.6. However, we do agree that it would be good to look at slower diffusing attractants too, so, we have now also performed 20 additional sets of simulations with diffusion speeds of only 10 micron^2/s for either attractant, autoattractant, or both, as well as combinations of diffusion speeds. We have added these to figure 6 and supplemental figures S2, S3, S4 and S5, and discuss them in the results where relevant.

The autoattractant is "produced by the cells in proportion to the amount of primary attractant they consume (Fig. 1D). The ratio of production is determined by the parameter settings of specific simulations. " This is not that clear. What is this ratio? Do you mean rate? And where can one find this parameter in the tables? Similarly, what does the x-axis of Fig. 2C mean (i.e. what does 100 or 0
mean?).

We did mean ratio, as the cells produce alpha units of autoattractant for every unit of attractant consumed. So 0 means no autoattractant is produced, and 100 means that 100 units of autoattractant are produced for every unit of attractant broken down.This makes the strength of the autoattractant signal depend on the strength of the attractant signal. This has now been clarified in section 2.1 and in the methods. As we vary this parameter in every set of simulations, there is no baseline parameter to list in the table, but we have now also included it in the table as "variable" for completeness.

Did I understand correctly that the chemotactic response is identical for both the attractant and the autoattractant? How realistic is this?

It is assumed to be equal in the model, but in many ways the response can be rescaled freely. For example, for a decayed autoattractant, if the kd and production are rescaled by the same constant, behaviour is identical. We have scaled them to be the same magnitude in this paper for ease of visualisation. We now clarify this in the discussion. We do assume that all receptors are weighed equally in making chemotactic decisions, so that there is no intrinsic preference for autoattractant over primary attractant or vice versa. We now include variations on this as a possible future extension in the discussion.

I am not sure one needs Fig. 3B. Fig. 3A already shows the main result and drawing a band with sharp boundaries is subjective.

We put this figure in as we found that some of our proofreaders were not familiar with heatmaps, and needed some more guidance in how to interpret them. For this reason we tend towards keeping it, but if the referee still disagrees strongly we will happily remove it.

"We showed how a narrow range of autoattractant lifetimes " This is a subjective statement. For 1000s, a fast-moving cell has traveled ~330 micron, a significant fraction of the chamber. So this upper limit is not that surprising.
We considered this to be a narrow range within the large range of autoattractant lifetimes we observed in figure 3F. It is indeed not surprising that there is an upper limit of useful lifetimes, but we did consider it important to note that the most useful lifetime was the same across the conditions we tested, as this provided the basis for our further investigation into imposing specific lifetimes. We have now reworded it to say "similar autoattractant lifetimes are optimal across environments"

Fig. 4F and G are not clear to me. What does "baseline conditions of Fig. 3A compared to those of 3C,D, and E" and "baseline conditions of Fig. 4B compared to those of 4C,D, and E" mean? Please explain better.

In short, each dot represents a combination of autoattractant production and autoattractant lifetime. For each dot, the migration with the baseline conditions places the x position, and migration in a different condition (color-coded) sets the y-position. We have now clarified this in the text and in the figure caption.
