# Supplementary material for: Communication through autoattractants can enhance and limit collective migration of immune cells
Source: PLoS Comput Biol. 2026 Sep 22;22(9):e1014809. doi: 10.1371/journal.pcbi.1014809 (PMC13626474; doi:10.1371/journal.pcbi.1014809)
Supplement: Peer review history

# Peer review: Decision Letter 0

**DOI:** 10.1371/journal.pcbi.1014809.r001

**Authors:** Boyce Griffith, Calina Copos

7 Jul 2026

Tell your friends: communication through autoattractants can enhance and limit migration of immune cells

PLOS Computational Biology

Dear Dr. Versluis,

Thank you very much for submitting your manuscript to PLOS Computational Biology. After careful consideration, we feel that it has merit and is an appropriate fit for the journal but does not fully meet PLOS Computational Biology's publication criteria as it currently stands. Therefore, we kindly invite you to submit a revised version of the manuscript that addresses the points raised during the review process.

Please submit your revised manuscript by Sep 05 2026 11:59PM. If you will need more time than this to complete your revisions, please reply to this message or contact the journal office at ploscompbiol@plos.org. When you're ready to submit your revision, log on to https://www.editorialmanager.com/pcompbiol/ and select the 'Submissions Needing Revision' folder to locate your manuscript file.

Please include the following items when submitting your revised manuscript:

* A letter that responds to each point raised by the editor and reviewer(s). You should upload this letter as a separate file labeled 'Response to Reviewers'. This file does not need to include responses to formatting updates and technical items listed in the 'Journal Requirements' section below.

* A marked-up copy of your manuscript that highlights changes made to the original version. You should upload this as a separate file labeled 'Revised Manuscript with Track Changes'.

* An unmarked version of your revised paper without tracked changes. You should upload this as a separate file labeled 'Manuscript'.

Kind regards,

Calina Copos, Ph.D.

Academic Editor

PLOS Computational Biology

Boyce Griffith

Section Editor

PLOS Computational Biology

**Additional Editor Comments (if provided):**

**Journal Requirements:**

If the reviewer comments include a recommendation to cite specific previously published works, please review and evaluate these publications to determine whether they are relevant and should be cited. There is no requirement to cite these works unless the editor has indicated otherwise.

1) Please ensure that the CRediT author contributions listed for every co-author are completed accurately and in full.

At this stage, the following Authors/Authors require contributions: David M. Versluis, and Robert H. Insall. Please ensure that the full contributions of each author are acknowledged in the "Add/Edit/Remove Authors" section of our submission form.

The list of CRediT author contributions may be found here: https://journals.plos.org/ploscompbiol/s/authorship#loc-author-contributions

2) We ask that a manuscript source file is provided at Revision. Please upload your manuscript file as a .doc, .docx, .rtf or .tex. If you are providing a .tex file, please upload it under the item type u2018LaTeX Source Fileu2019 and leave your .pdf version as the item type u2018Manuscriptu2019.

3) Please upload all main figures as separate Figure files in .tif or .eps format. For more information about how to convert and format your figure files please see our guidelines:

https://journals.plos.org/ploscompbiol/s/figures

4) Please upload a copy of Figures FIG 1 A-F, 2A-E, 3A-F, 4A-G, 5A-E, and 6A-E which you refer to in your text on pages 3, 4, 6, 8, and 11. Or, if the figure is no longer to be included as part of the submission please remove all reference to it within the text.

5) We have noticed that you have uploaded Supporting Information files, but you have not included a list of legends. Please add a full list of legends for your Supporting Information files after the references list.

6) We notice that your supplementary Figures are included in the manuscript file. Please remove them and upload them with the file type 'Supporting Information'. Please ensure that each Supporting Information file has a legend listed in the manuscript after the references list.

7) Please amend your detailed Financial Disclosure statement. This is published with the article. It must therefore be completed in full sentences and contain the exact wording you wish to be published.

i) State the initials, alongside each funding source, of each author to receive each grant. For example: "This work was supported by the National Institutes of Health (####### to AM; ###### to CJ) and the National Science Foundation (###### to AM)."

ii) State what role the funders took in the study. If the funders had no role in your study, please state: "The funders had no role in study design, data collection and analysis, decision to publish, or preparation of the manuscript."

iii) If any authors received a salary from any of your funders, please state which authors and which funders..

If you did not receive any funding for this study, please simply state: u201cThe authors received no specific funding for this work.u201d

8)We have amended your Competing Interest statement to comply with journal style. We kindly ask that you double check the statement and let us know if anything is incorrect

**Reviewers' comments:**

Reviewer's Responses to Questions

**Comments to the Authors:**

**Please note here if the review is uploaded as an attachment.**

Reviewer #1: This is a novel and useful computational study of collective cell chemotaxis in the

presence of an 'autoattractant' that cells emit and respond to, in addition to common

external chemoattractant. The authors simulate intuitive hybrid model in which the cells

are discrete traveling agents, while the chemicals are simulated according to

reaction-diffusion models. The main finding of the paper is that there are certain

optimal regimes in which the autoattractant dynamics have to be fine-tuned in order

to maximize the response to the external chemoattractant.

The paper is clearly written, accurate and well fit for PLoS Comp Biol.

I suggest several minor changes:

1) What I was missing in the text is mechanistic explanations of the results of the numerical experiments. For example, "too much autoattractan --> worse performance" is just stated plainly.

There has to be a simple explanations, like "in this regime cells aggregate too much and then..."

maybe their movement is hindered, or they degrade the external chemoattractant too much, or...

I strongly suggest to write such interpretations after every result of the numerical experiment,

and then summarize them in the Discussion.

2) Numbers in Tables 1 and 2 are told to be coming from published data. Need references for

these parameters' values, with explanations - was this or that parameter measured, or coming

from some indirect inference, or... And I imagine that at least some of these parameters are

from some kinds of fits - basically, need details.

3) "Tell your friends:" part of the title is cute, but perhaps not a good idea for a research

paper (it would be fine and even welcome for a review)

Reviewer #2: This paper builds on the group's work on chemotaxis through self-generated gradients. A new ingredient is introduced: cells not only consume an attractant but also produced an autoattractant.

The paper then determines for which conditions more cells can reach the half-point of the chamber.

I believe the article is not always as clear as it could be. I have some specific comments below. My most serious comment,however, is that it is unclear if this scenario is actually at play in vivo.

The authors acknowledge that autoattractants "would be short-lived, it is inherently difficult to find additional autoattractants in vitro or in vivo: samples collected would be depleted of autoattractant

before measurements could be made. New techniques may find autoattractants in many more cell types." This, combined with the assumptions make in the study (constant speed, cells response identical to both attractant and autoattractant, etc.) dampens my enthusiasm for the paper. However, the results are scientifically sound and I urge the authors to make a better argument why this scenario is plausible.

The diffusion constant is taken to be 100 micron^2/s and the timestep is 6s. This means that in one timestep, the attractant can diffuse over a length scale of sqrt(600)~25 micron. I could not find the size of the computational cell but this seems to me very large compared to a, e.g., neutrophil.

The autoattractant is "produced by the cells in proportion to the amount of primary attractant they consume (Fig. 1D). The ratio of production is determined by the parameter settings of specific simulations. " This is not that clear. What is this ratio? Do you mean rate? And where can one find this parameter in the tables? Similarly, what does the x-axis of Fig. 2C mean (i.e. what does 100 or 0

mean?).

Did I understand correctly that the chemotactic response is identical for both the attractant and the autoattractant? How realistic is this?

I am not sure one needs Fig. 3B. Fig. 3A already shows the main result and drawing a band with sharp boundaries is subjective.

"We showed how a narrow range of autoattractant lifetimes " This is a subjective statement. For 1000s, a fast-moving cell has traveled ~330 micron, a significant fraction of the chamber. So this upper limit is not that surprising.

Fig. 4F and G are not clear to me. What does "baseline conditions of Fig. 3A compared to those of 3C,D, and E" and "baseline conditions of Fig. 4B compared to those of 4C,D, and E" mean? Please explain better.

**********

**Have the authors made all data and (if applicable) computational code underlying the findings in their manuscript fully available?**

The PLOS Data policy requires authors to make all data and code underlying the findings described in their manuscript fully available without restriction, with rare exception (please refer to the Data Availability Statement in the manuscript PDF file). The data and code should be provided as part of the manuscript or its supporting information, or deposited to a public repository. For example, in addition to summary statistics, the data points behind means, medians and variance measures should be available. If there are restrictions on publicly sharing data or code —e.g. participant privacy or use of data from a third party—those must be specified.

Reviewer #1: Yes

Reviewer #2: Yes

**********

PLOS authors have the option to publish the peer review history of their article (what does this mean?). If published, this will include your full peer review and any attached files.

If you choose “no”, your identity will remain anonymous but your review may still be made public.

**Do you want your identity to be public for this peer review?**  For information about this choice, including consent withdrawal, please see our Privacy Policy.

Reviewer #1: No

Reviewer #2: No

[NOTE: If reviewer comments were submitted as an attachment file, they will be attached to this email and accessible via the submission site. Please log into your account, locate the manuscript record, and check for the action link "View Attachments". If this link does not appear, there are no attachment files.]

**Figure resubmission:**

**Reproducibility:**

?>

# Author comment: Author response to Decision Letter 1

**DOI:** 10.1371/journal.pcbi.1014809.r002

1 Sep 2026

Attachment Submitted filename: reviewer response.pdf

# Peer review: Decision Letter 1

**DOI:** 10.1371/journal.pcbi.1014809.r003

**Authors:** Boyce Griffith, Calina Copos

7 Sep 2026

Dear Dr. Versluis,

We are pleased to inform you that your manuscript 'Communication through autoattractants can enhance and limit collective migration of immune cells' has been provisionally accepted for publication in PLOS Computational Biology.

Before your manuscript can be formally accepted you will need to complete some formatting changes, which you will receive in a follow up email. A member of our team will be in touch with a set of requests.

Please note that your manuscript will not be scheduled for publication until you have made the required changes, so a swift response is appreciated.

IMPORTANT: The editorial review process is now complete. PLOS will only permit corrections to spelling, formatting or significant scientific errors from this point onwards. Requests for major changes, or any which affect the scientific understanding of your work, will cause delays to the publication date of your manuscript.

Should you, your institution's press office or the journal office choose to press release your paper, you will automatically be opted out of early publication. We ask that you notify us now if you or your institution is planning to press release the article. All press must be co-ordinated with PLOS.

Thank you again for supporting Open Access publishing; we are looking forward to publishing your work in PLOS Computational Biology.

Best regards,

Calina Copos, Ph.D.

Academic Editor

PLOS Computational Biology

Boyce Griffith

Section Editor

PLOS Computational Biology

***********************************************************

Reviewer's Responses to Questions

**Comments to the Authors:**

**Please note here if the review is uploaded as an attachment.**

Reviewer #1: I am satisfied with the revisions

Reviewer #2: The authors have addressed all comments

**********

**Have the authors made all data and (if applicable) computational code underlying the findings in their manuscript fully available?**

The PLOS Data policy requires authors to make all data and code underlying the findings described in their manuscript fully available without restriction, with rare exception (please refer to the Data Availability Statement in the manuscript PDF file). The data and code should be provided as part of the manuscript or its supporting information, or deposited to a public repository. For example, in addition to summary statistics, the data points behind means, medians and variance measures should be available. If there are restrictions on publicly sharing data or code —e.g. participant privacy or use of data from a third party—those must be specified.

Reviewer #1: Yes

Reviewer #2: Yes

**********

PLOS authors have the option to publish the peer review history of their article (what does this mean?). If published, this will include your full peer review and any attached files.

If you choose “no”, your identity will remain anonymous but your review may still be made public.

**Do you want your identity to be public for this peer review?**  For information about this choice, including consent withdrawal, please see our Privacy Policy.

Reviewer #1: No

Reviewer #2: No

# Editor report: Acceptance letter

**DOI:** 10.1371/journal.pcbi.1014809.r004

**Authors:** Boyce Griffith, Calina Copos

PCOMPBIOL-D-26-01296R1

Communication through autoattractants can enhance and limit collective migration of immune cells

Dear Dr Versluis,

I am pleased to inform you that your manuscript has been formally accepted for publication in PLOS Computational Biology. Your manuscript is now with our production department and you will be notified of the publication date in due course.

The corresponding author will soon be receiving a typeset proof for review, to ensure errors have not been introduced during production. Please review the PDF proof of your manuscript carefully, as this is the last chance to correct any errors. Please note that major changes, or those which affect the scientific understanding of the work, will likely cause delays to the publication date of your manuscript.

Soon after your final files are uploaded, unless you have opted out, the early version of your manuscript will be published online. The date of the early version will be your article's publication date. The final article will be published to the same URL, and all versions of the paper will be accessible to readers.

For Research, Software, and Methods articles, you will receive an invoice from PLOS for your publication fee after your manuscript has reached the completed accept phase. If you receive an email requesting payment before acceptance or for any other service, this may be a phishing scheme. Learn how to identify phishing emails and protect your accounts at https://explore.plos.org/phishing.

Thank you again for supporting PLOS Computational Biology and open-access publishing. We are looking forward to publishing your work!

With kind regards,

Sharmila Kamatchi

PLOS Computational Biology | Carlyle House, Carlyle Road, Cambridge CB4 3DN | United Kingdom ploscompbiol@plos.org | Phone +44 (0) 1223-442824 | ploscompbiol.org | @PLOSCompBiol
